# The Psychosocial Impact of Self-Reported Morning Allergy Symptoms: Findings from an Australian Internet-Based Survey

**DOI:** 10.1155/2010/710926

**Published:** 2010-06-08

**Authors:** Timothy J. Sharp, Celina Seeto

**Affiliations:** ^1^RMIT School of Health Sciences, Melbourne, VIC 3083, Australia; ^2^Happiness Institute, Suites 101/102, 74 Pitt Street, Sydney NSW 2000, Australia; ^3^Faculty of Pharmacy, Building A15, The University of Sydney, Sydney NSW 2006, Australia; ^4^Schering-Plough Pty Limited, Level 4, 66 Waterloo Road, North Ryde NSW 2113, Australia

## Abstract

*Background*. Allergies can substantially impact health-related quality of life (HRQL). We investigated the psychosocial impact of morning symptoms amongst Australian adults with self-reported allergic rhinitis (AR). *Method*. An online survey comprising 24 questions was conducted in August 2008. Inclusion criteria were age (20–49 years) and self-reported moderate to severe symptoms of AR. *Results*. One thousand sixty respondents met the inclusion criteria. Amongst consumers with self-reported AR, symptoms were more severe in the morning in 597 (56%) and affected mood in 1025 (97%). Nine hundred seventy (91%) indicated that their symptoms had some impact on their day ahead and 868 (82%) reported a negative impact on relationships. Morning symptoms in particular had a substantial affect on mood for the day. HRQL impact was more pronounced in those who reported severe symptoms and in females. *Discussion*. Encouraging consumers with self-diagnosed AR to seek formal diagnosis and offering appropriate treatment strategies, such as those offering sustained effectiveness over 24-hours, may aid in negating the negative impact of morning symptoms.

## 1. Introduction

Currently one in five (4.1 million) Australians have at least one allergy; with prevalence estimated to be growing at a rate of 0.09% per annum [1]. Most suffer from more than one condition at a time (an average of 1.74 comorbid allergies per person) and over half (51.4%) are 20–49 years of age [1]. 

The prevalence of allergic rhinitis is increasing; approximately 16% of Australians have allergic rhinitis, including about 19% of working aged adults [2]. The relative risk of mortality due to allergic rhinitis is low [1]; explaining why it has previously been regarded as a trivial condition. However, it is widely accepted that allergic rhinitis can have a significant negative impact on several areas of health-related quality of life (HRQL) [3–5]. Daily activities are impaired in more than 80% of patients with moderate or severe allergic rhinitis and 40% of those with mild disease [6]. Health impairment often leads to work impairment in the form of both absenteeism and presenteeism. The ability of allergic rhinitis sufferers to engage in productive work has been shown to be influenced by sleep, HRQL, specific symptoms, and antihistamine use [7]. Our survey examined the impact of self-reported allergic rhinitis on the emotional and psychological wellbeing of Australian adults.

## 2. Methods

In August 2008, an online survey was conducted amongst participants from a permission based panel (Galaxy DP Pty Ltd, Australia). In a permission based panel, all panellists give prior permission to receive email invitations to participate in research. With each invitation they have the opportunity to accept or decline and are paid $2.00 to participate in each survey. A minimum sample size of 200 participants per state was sought to ensure a representative sample. The total sample was then weighted to national population proportions (issued by the Australian Bureau of Statistics (ABS)) based on state, age, and gender.

The survey tool was not validated, but was based on that previously used by Long in a similarly conducted research carried out in the US in 2005 [8]. The survey comprised five screening questions, six demographic questions and 13 questions relating to the respondents' self-reported experience of allergies and their subsequent impact on quality of life. The questionnaire was administered in English only. No identifying data were collected. 

Male and female respondents, aged 20 to 49 years, were included if they had self-reported moderate to severe hay fever, hives/skin allergies, or allergies triggered by air-borne factors (pet dander, pollen/grass, dust mites, mould). In addition, patients were asked to report “How often they suffer from allergies?” The possible options for this question were based on the ARIA guideline classification of intermittent versus persistent allergic rhinitis [9]. The options listed corresponded to the classification as such: intermittent allergic rhinitis = less than four days a week *and* seasonally for less than four weeks at a time and persistent allergic rhinitis = more than four days a week *and* seasonally for more than four weeks at a time.

Respondents were excluded if they had self-reported mild allergies, only asthma, or had allergies triggered by any factors other than those listed above.

Participants' responses were summarised and analysed to identify any significant differences between groups. Statistical analysis was conducted using *z* tests for the difference between proportions.

## 3. Results

### 3.1. Respondent Characteristics

One thousand, four hundred fifty three people responded to the survey, of whom 1060 met the study criteria, completed the survey and were included in the study results. Overall demographic characteristics are listed in Table 1. 

Of the respondents who met the study criteria, 82% (*n* = 865) self-reported having “moderate” symptoms and 18% (*n* = 195) self-reported having “severe” symptoms. The most frequently self-reported type of allergy was hayfever (*n* = 879), followed by pollen/grass allergies (*n* = 665), hives/skin allergies (*n* = 450), dust mite allergies (*n* = 420), pet allergies (*n* = 399), and mould allergies (*n* = 199).

### 3.2. Relationship of Self-Reporting with ARIA Classification

Of the respondents who self-reported as having “moderate” symptoms, 55% (*n* = 479/865) stated having allergy symptoms that would be classified as being persistent allergic rhinitis sufferers. Whereas, 76% (*n* = 148/195) of those who reported having “severe” allergy symptoms would be classified as being persistent allergic rhinitis sufferers.

### 3.3. Assessment of Allergy Symptoms

Of the respondents who reported having moderate symptoms, 54% (*n* = 466/865) reported that they “always or mostly” used medication to treat their allergy symptoms, whilst 46% (*n* = 399/865) reported that they “sometimes” used medications to treat their allergy symptoms. This is in contrast to those who reported having severe symptoms, where 82% (*n* = 159/195) reported that they “always/mostly” used medication and 19% (*n* = 36/195) reported that they “sometimes” used medication.

**Figure 1:**
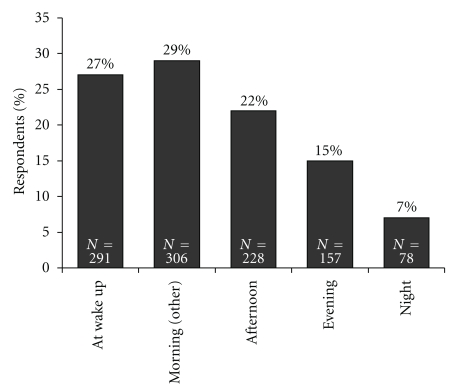
Time when allergy symptoms are most severe. Percentage of respondents who replied to the question “*When are your allergy symptoms most severe*?”

Over half of all the participants (597; 56%) reported that their allergy symptoms were more severe in the morning than at any other time of the day (Figure 1). There was no discernable difference between those who reported having “moderate” (55%, *n* = 477/875) or “severe” (62%, *n* = 120/185) symptoms. More females reported that their allergy symptoms were at their worst in the mornings (Females: 60% [328/545] versus Males: 52% [269/515]; *P* < .01). 

When asked which symptoms were worse in the morning, sneezing (630; 59%), itchy/watery eyes (621; 59%), running nose (542; 51%), and blocked nose (459, 43%) were the most frequently reported. “*Runny nose*” (630; 63%) and “*blowing nose*” (610; 58%) were the most frequently reported symptoms which made respondents feel embarrassed or unattractive around others.

### 3.4. Assessment of Psychosocial Impact

The majority of respondents reported that their symptoms had an impact on their lives, either by waking them up (88%; *n* = 931/1060) or by affecting their mood for the day (97%; *n* = 1025/1060). When asked how their symptoms affected their mood for the day (Table 2), being “*irritable*” and being “*tired and exhausted*” were most commonly reported. Almost all respondents (970; 91%) indicated that their symptoms had some impact on their day ahead, 868 (82%) reporting a negative impact on relationships and/or family life, and 969 (91%) reporting that their symptoms had prevented them from doing something they would usually have done (e.g., exercised or gone to work).

With the exception of reporting being “*tired and exhausted,*” those with self-reported severe allergy symptoms had a significantly higher impact on their mood compared to those with self-reported moderate allergy symptoms (*P* < .05). Significantly more females than males expressed that their symptoms made them “*irritable,*” “*tired and exhausted*”, and “*depressed*” (*P* < .05 for each measure; Table 2). A similar trend was seen with responses to the impact on the day ahead (Table 2). Females reported a significantly higher impact than males for several measures relating to appearance and or interpersonal interactions. In contrast, males were significantly more likely to report that their professionalism at work would be compromised (24% [125/515] versus 17% [93/545], *P* < .05). 

**Figure 2:**
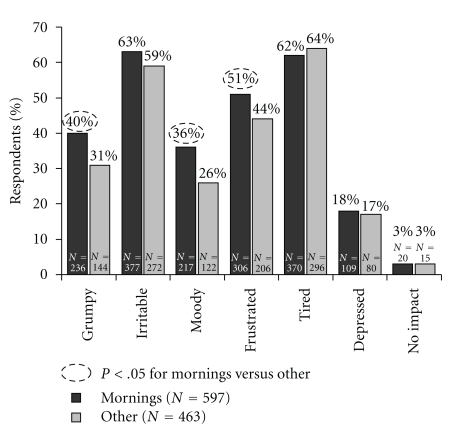
Impact of morning allergy symptoms on mood for the day. Percentage of respondents who replied to the question “*When you get allergies, how does it affect your mood for the day*?”

**Figure 3:**
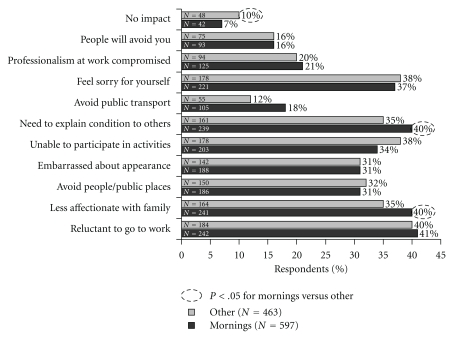
Impact of morning allergy symptoms on the day ahead. Percentage of respondents who replied to the question “*What impact do your allergies have on your day ahead*?”

The impact of morning symptoms on mood was analysed by the time of day that the symptoms were most severe (Figure 2). Respondents who reported that their symptoms were most severe during the morning were more likely to report that their condition made them “*grumpy,*” “*moody*,” and “*frustrated*.” Conversely, people who suffered more at other times of the day (including the night time) were more likely to report feeling “*irritable*” and “*tired and exhausted.*” The negative impact of morning allergy symptoms also extended to how people behaved during the day (Figure 3), with a significant impact on usual morning behavioural patterns (“*dreading public transport,*” “*feeling the need to explain that you are not sick*”, and “*being less affectionate with partner and children*”).

## 4. Discussion

Much work has already been conducted to determine the impact of allergic rhinitis on HRQL, however, most is focussed on individuals with verified allergy leaving out those with self-reported allergy symptoms. Our study has shown that self-reported symptoms of moderate to severe allergic rhinitis have a significant negative impact on emotional and psychological wellbeing. Almost two out of every three respondents reported being either “*irritated*” or “*tired and exhausted*” as a consequence of their symptoms. Morning symptoms, in particular, have a substantial impact on mood for the day (sufferers report feeling more “*grumpy*,” “*moody,*” and “*frustrated*”) as well as on their usual daily activities. These findings are similar to those published previously, which demonstrated that morning allergy symptoms not only have a negative impact on the quality of life of individuals with moderate to severe allergic rhinitis but that that these symptoms substantially impact the person for the rest of the day [8]. The results obtained in our study have extended these findings, demonstrating how symptoms in people who report self-awareness of allergic rhinitis negatively impact on home and work life and relationships with partners.

Both seasonal and perennial allergic rhinitis sufferers may develop a group of psychological complaints related to symptom severity [10]. Perceptions of symptom severity have been shown to be positively associated with personality factors (hypochondriasis and somatic awareness) whilst being independent of skin prick test results [11]. Neuroticism, the tendency to experience negative emotions and to be highly self-conscious and somatically concerned, has been positively correlated with allergy [12]. Furthermore, findings suggest that less adaptive coping strategies are associated with higher levels of psychological distress amongst allergy sufferers [13]. 

Gender differences were apparent in our study, particularly in areas of the questionnaire related to appearance. This could be explained by sociocultural attitudes, females have a greater willingness to acknowledge and report symptoms. Gender-specific differences in the interpretation of symptoms and their impact on the individual have previously been reported [14, 15]. Moreover, gender is known to play a strong role in depression and other psychological disorders. Amongst a sample of self-reported adult allergy sufferers in the US, there was a significant relationship between allergy and depression in women but not in men [12]. In the attempt to better understand the links between allergy and psychological status it is still not clear which comes first. 

The most notable limitation of our study is that the questionnaire used was not formally validated. The questions were developed based on review of prior published instruments and conformed closely to that utilised by Long [8]. Our questionnaire extended focused predominantly on the emotional and psychological impact of typical allergic rhinitis symptoms. As such, our questionnaire had an increased focus on how the symptoms made the respondent feel and which particular symptoms made them feel embarrassed or unattractive while at work or in the presence of others. The absence of a numerical response scale in the questionnaire limits the ability to compare our results with those obtained in other studies. Although the sample sizes were weighted with respect to ABS data, a permission-based panel was used such that the study population may not be representative of the general allergy population. 

Nasal congestion has been described as “*a cardinal symptom of allergic rhinitis*” [16]. In our study only 43% of respondents reported that nasal congestion was worse in the morning, which is lower than was expected. One explanation for this discrepancy is that it is a direct result of a problem with the data collection tool. The question asked was “*For you, which allergy symptoms are worse in the mornings*?” It may be that nasal congestion is always present and therefore does not get worse in the mornings. Alternatively, our result may simply reflect that the study respondents did not have to have a prior physician diagnosis of allergy. This is consistent with prior research; it has previously been shown that nasal congestion is the symptom most likely to prompt a person with allergic rhinitis to seek medical help [17] and that “blocked nose” is not the dominant symptom in people who self-report awareness of allergic rhinitis [18]. 

The widespread availability of nonprescription allergy medications means that many people with symptoms suggestive of allergic rhinitis self-diagnose their condition and present to the pharmacy for treatment. In many cases they are then able to self-select a medication without recourse to health professional advice. It has previously been established that self-management of allergy symptoms is enhanced when healthcare professional intervention is sought and tailored goals established [19]. We have shown that symptoms, particularly those that are most severe in the mornings, can substantially impact the overall emotional and psychological wellbeing of consumers who are self-aware of allergic rhinitis and who are self-medicating. Encouraging these consumers to seek formal diagnosis and then combining appropriate treatment strategies, such as those offering sustained effectiveness over 24-hours, with an individually tailored, goal-oriented, and self-management program may aid in optimising symptom management.

## Figures and Tables

**Table 1:** Demographic characteristics of respondents with self-reported allergic rhinitis (*N* = 1060)*.

Characteristic	Number (%)
Gender	
Male	515 (49)
Female	545 (51)

Age (years)	
20–29	381 (36)
30–39	355 (33)
40–49	325 (31)

Geographical location	
New South Wales/Australian Capital Territory	371 (35)
Victoria/Tasmania	293 (28)
Queensland	210 (20)
South Australia	79 (7)
Western Australia	107 (10)

Marital status	
Married	460 (43)
De facto/Living together	244 (23)
Separated, widowed, or divorced	76 (7)
Never married	280 (26)

Children	
Yes	570 (54)
No	490 (46)

Work status	
Full time	657 (62)
Part time	176 (17)
Full time student	78 (7)
Not working/studying	150 (14)

*Weighted sample. The sample was weighted to reflect Australian Bureau of Statistics estimates based on state, age, and gender. The actual number of respondents from each state was 215 (NSW/ACT); 219 (VIC/TAS); 220 (QLD); 206 (SA); 200 (WA).

**Table 2:** Impact of self-reported allergies on mood for the day and the day ahead.

	All respondents *N* (%)	Gender *N* (%)		Symptom severity *N* (%)	
Characteristic		Male	Female	Severe	Moderate
“When you get allergies, how does it affect your mood for the day?”					
Grumpy	380 (36)	184 (36)	196 (36)	**99 (51)^‡^**	281 (32)
Irritable	650 (61)	301 (58)	349 (64)*	**129 (66)^‡^**	521 (60)
Moody	340 (32)	174 (34)	166 (30)	**87 (44)^‡^**	253 (29)
Frustrated	511 (48)	237 (46)	274 (50)	**106 (54)^‡^**	406 (47)
Tired and exhausted	666 (63)	305 (59)	361 (66)*	128 (66)	537 (62)
Depressed	188 (18)	81 (16)	107 (20)*	**53 (27)^‡^**	135 (16)
No impact	35 (3)	18 (3)	17 (3)	10 (5)	26 (3)

“What impact do your allergies have on your day ahead?”					
Reluctant to go to work	426 (40)	221 (43)	205 (38)*	80 (41)	346 (40)
Less affectionate with partner or children	405 (38)	197 (38)	208 (38)	**101 (52)^‡^**	304 (35)
Avoid people/public places	335 (32)	142 (28)	193 (35)*	**76 (39)^‡^**	259 (30)
Embarrassed about appearance	329 (31)	140 (27)	190 (35)*	68 (35)	262 (30)
Unable to participate in everyday activities	380 (36)	191 (37)	189 (35)	**82 (42)^‡^**	298 (34)
Need to explain that you are not sick	400 (38)	168 (33)	232 (43)*	**87 (45)^‡^**	313 (36)
Dread taking public transport	160 (15)	72 (14)	87 (16)	33 (17)	127 (15)
Feel sorry for yourself	398 (38)	158 (31)	240 (44)*	**89 (46)^‡^**	309 (36)
Professionalism will be compromised	219 (21)	**125 (24)^†^**	93 (17)	**52 (26)^‡^**	167 (19)
People will avoid you	168 (16)	74 (14)	94 (17)	**39 (20)^‡^**	129 (15)
None of the above	90 (9)	50 (10)	40 (7)	13 (7)	77 (9)

*P < .05 for females versus males; ^†^ *P* < .05 for males versus females; ^‡^ *P* < .05 for people with severe versus moderate symptoms.

## References

[1] Access Economics Australasian Society of Clinical Immunology and Allergy. The economic impact of allergic disease in Australia: not to be sneezed. http://www.accesseconomics.com.au/publicationsreports/getreport.php?report=158&id=205.

[2] Australian Institute of Health and Welfare (2006). *Australia’s Health 2006*.

[3] Nathan RA (2007). The burden of allergic rhinitis. *Allergy and Asthma Proceedings*.

[4] Camelo-Nunes IC, Solé D (2010). Allergic rhinitis: indicators of quality of life. *Jornal Brasileiro de Pneumologia*.

[5] Meltzer EO, Nathan R, Derebery J (2009). Sleep, quality of life, and productivity impact of nasal symptoms in the United States: findings from the Burden of Rhinitis in America survey. *Allergy and Asthma Proceedings*.

[6] Bousquet J, Neukirch F, Bousquet PJ (2006). Severity and impairment of allergic rhinitis in patients consulting in primary care. *Journal of Allergy and Clinical Immunology*.

[7] Szeinbach SL, Seoane-Vazquez EC, Beyer A, Williams PB (2007). The impact of allergic rhinitis on work productivity. *Primary Care Respiratory Journal*.

[8] Long AA (2007). Findings from a 1000-patient internet-based survey assessing the impact of morning symptoms on individuals with allergic rhinitis. *Clinical Therapeutics*.

[9] ARIA ARIA: at a glance pocket guide. http://www.whiar.org/docs/ARIA_PG_08_View_WM.pdf.

[10] Bavbek S, Kumbasar H, Tugcu H, Misirligil Z (2002). Psychological status of patients with seasonal and perennial allergic rhinitis. *Journal of Investigational Allergology and Clinical Immunology*.

[11] Graif Y, Goldberg A, Tamir R, Vigiser D, Melamed S (2006). Skin test results and self-reported symptom severity in allergic rhinitis: the role of psychological factors. *Clinical and Experimental Allergy*.

[12] Goodwin RD, Castro M, Kovacs M (2006). Major depression and allergy: does neuroticism explain the relationship?. *Psychosomatic Medicine*.

[13] Knibb RC, Horton SL (2008). Can illness perceptions and coping predict psychological distress amongst allergy sufferers?. *British Journal of Health Psychology*.

[14] Osman M, Hansell AL, Simpson CR, Hollowell J, Helms PJ (2007). Gender-specific presentations for asthma, allergic rhinitis and eczema in primary care. *Primary Care Respiratory Journal*.

[15] Marklund B, Ahlstedt S, Nordström G (2004). Health-related quality of life among adolescents with allergy-like conditions—with emphasis on food hypersensitivity. *Health and Quality of Life Outcomes*.

[16] Nathan RA (2008). The pathophysiology, clinical impact, and management of nasal congestion in allergic rhinitis. *Clinical Therapeutics*.

[17] Shedden A (2005). Impact of nasal congestion on quality of life and work productivity in allergic rhinitis: findings from a large online survey. *Treatments in Respiratory Medicine*.

[18] Bauchau V, Durham SR (2004). Prevalence and rate of diagnosis of allergic rhinitis in Europe. *European Respiratory Journal*.

[19] O’Connor J, Seeto C, Saini B (2008). Healthcare professional versus patient goal setting in intermittent allergic rhinitis. *Patient Education and Counseling*.

